# Genetic diversity of *Mycobacterium leprae* in the state of São Paulo, an area of low-leprosy incidence in Brazil

**DOI:** 10.1590/0037-8682-0612-2022

**Published:** 2023-03-27

**Authors:** Amanda Juliane Finardi, Nathan Guilherme de Oliveira, Eloise Brasil de Moraes, Lavínia Cássia Ferreira Batista, Bruna Eduarda Bortolomai, Philip Noel Suffys, Ida Maria Foschiani Dias Baptista

**Affiliations:** 1 Instituto Lauro de Souza Lima, Bauru, SP, Brasil.; 2 Universidade Estadual Paulista, Faculdade de Medicina, Botucatu, SP, Brasil.; 3 Fundação Oswaldo Cruz, Laboratório de Biologia Molecular Aplicada a Micobactérias, Rio de Janeiro, RJ, Brasil.

**Keywords:** Leprosy, Transmission, Mycobacterium leprae, Genetic Diversity, Geographic Information System

## Abstract

**Background:**

Brazil has the second largest number of leprosy cases worldwide, and the state of São Paulo has been considered non-endemic since 2006.

**Methods:**

We analyzed 16 variable number tandem repeats loci and three single nucleotide polymorphisms loci of *Mycobacterium leprae (M. leprae)* in 125 clinical isolates from patients in different municipalities in the state.

**Results:**

The clustering pattern of *M. leprae* indicated that the transmission of leprosy persisted in the state and included scenarios of intra-extra-familial transmission in areas with low endemicity.

**Conclusions:**

A significantly active circulation of *M. leprae* was observed. Therefore, surveillance and control measures must be implemented.

Leprosy is a chronic and systemic disease caused by *Mycobacterium leprae (M. leprae)* or *Mycobacterium lepromatosis* and persists as a public health problem in Brazil. The disease has a slow, progressive evolution and, in the absence of early diagnosis and treatment, can cause deformities and irreversible physical disabilities^1^.

After the introduction of standardized multidrug therapy (MDT) in the state of São Paulo, southeastern Brazil, there has been a significant decrease in leprosy detection coefficients. From 2006 onward, the state was considered non-endemic, with a prevalence rate below 1 case per 10.000 inhabitants, reaching 0.23/10.000 in 2015^2^.

However, a study in the city of Jardinópolis demonstrated an unpreparedness to diagnose leprosy among primary care professionals, with 24 new cases diagnosed in 2015 (4.4/10.000 inhabitants), contradicting the supposed non-endemicity in the state^3^. In 2017, São Paulo reported 1.617 notified cases of leprosy, but 19.4% of the municipalities had not yet reached their elimination goals. However, in that same year, evidence of active transmission in São Paulo was supported by the detection of new cases in children under 15 years of age, as well as by the arrival of patients at the reference health services with severe forms of the disease^2^.

Advances in genotyping of *M. leprae* based on single nucleotide polymorphisms (SNP) and variable number tandem repeats (VNTR) have revolutionized the understanding of the origin(s) of the disease, migratory flows, and transmission patterns^4^. Through the selection of VNTRs and their association with SNP, it is possible to verify the regional transmission of specific genotypes, such as multi-case families. It is also possible to carry out cluster identification and monitoring, understand the geographical distribution of leprosy, and identify populations at risk^5^ ^-^ ^7^. 

This study aimed to investigate the nature of *M. leprae* genotypes in patients diagnosed in São Paulo between 2002 and 2013, focusing on population structure and cluster levels. 

A cross-sectional molecular surveillance study was performed using DNA samples from 125 patients with leprosy that had been submitted to dermatoneurological diagnosis at the outpatient clinic of the Lauro de Souza Lima Institute between 2001 and 2013. Sociodemographic and clinical information, such as age, sex, address, clinical form, and treatment, were obtained from medical records. The start date of symptoms was not available. Figure 1 shows that the patients were from different administrative health regions with high, medium, and low endemicity for leprosy.

**FIGURE 1:**
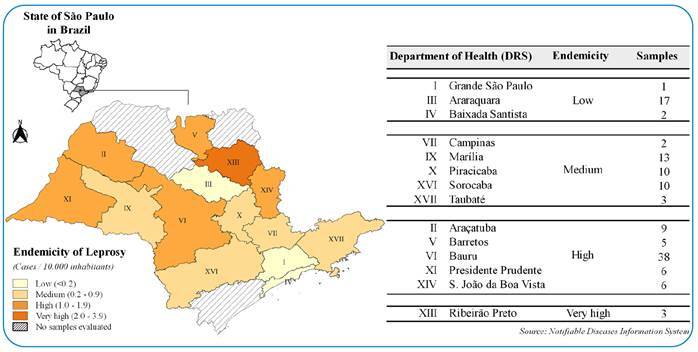
Regional health departments of the state of São Paulo, Brazil, according to endemicity and number of samples evaluated in the study.

Nucleic acids were extracted from biopsy specimens using a DNeasy Tissue Kit (Qiagen, Hilden, Germany). The multiplex PCR protocol consisted of four reactions for the amplification of 16 VNTR loci using fluorescent-labeled 5’: (i) AC8b, GTA9, GGT5, AT17, and 6-3a (or *rpo*T); (ii) 21-3, AC9, and AC8a; (iii) 27-5, 6-7, TA18, and TTC21; and (iv) 18-8, 12-5, 23-3, and TA10. Multiplex PCR products were separated using capillary electrophoresis (ABI 3130 Genetic Analyzer, Applied Biosystems) and fragment length analysis (FLA) was performed to determine the copy number of each VNTR locus using Peak Scanner software (version 1.0, Thermo Fisher Scientific)^7^. Differentiation between SNP types 1/2 and 3/4 was obtained by restriction analysis of the locus at nucleotide 2935685 by incubating 5 µL of PCR product with 5 units of BstUI (New England Biolabs, Beverly, MA) at 60 °C for 1 h. For further differentiation of SNP types 3 and 4, restriction analysis of nucleotide 14676 was performed by incubating with 5 Units of SmlI for 1 h at 50 °C. The restriction products were subjected to electrophoresis on a 3% agarose gel. The digestion indicated SNP type 4, while the lack of digestion identified SNP type 3^8^. To differentiate between types 1 and 2, sequencing of the SNP at position 1642875 was performed as described by Monot *et al*. (2005)^5^. Genotype similarity was analyzed using the categorical similarity index and unweighted pair group method with arithmetic mean (UPGMA) using Bionumerics Software (Version 7.6, Applied Maths, Belgium). The discriminatory power and allelic diversity were calculated using the h value and the Hunter Gaston discriminatory index (HGDI). VNTR loci were designated as “highly discriminant” (h > 0.6), “moderately discriminant” (0.3 ≤ h ≤ 0.6), and “poorly discriminant” (h < 0.3)^9^. The clustering rate (CR) was calculated for each method using the formula (nc − c)/n, where nc is the total number of isolates clustered by a given method, c is the number of clusters, and n is the total number of isolates in the dataset^10^. A cluster was defined as two or more isolates with identical VNTR patterns.

Using the Google Earth Pro 7.3.2 software, the addresses of the patients were transformed into geographic coordinates in the format degrees, minutes, and seconds (DDD°MM'SS. S") and imported as point layers into the open-source software Quantum GIS 3.14 (Qgis) for descriptive geospatial analysis. Shapefiles with the boundaries of the municipalities of São Paulo were imported from the Brazilian Institute of Geography and Statistics. DATUM SIRGAS 2000 was used as the basic cartographic coordinate system.

This study was approved by the Ethical Committee of the Lauro de Souza Lima Institute (No. 1.380.430). 

The patients were predominantly male (73.6%), self-reported as white (86.4%), had low education (24.1%), and had an average age of 49.5 years. According to the clinical classification, 63 patients (50.4%) were lepromatous, 33 (26.4%) were borderline lepromatous, 17 (13.6%) were borderline, 6 (4.8%) were borderline tuberculoid, and 2 (1.6%) were tuberculoid. Four patients (3.2%) were not classified.

The samples evaluated in this study represent only 0.5% of the total number of leprosy cases reported between 2001 and 2013 in São Paulo; however, they present a sociodemographic and clinical profile similar to that of other endemic regions of the country, that is, men of economically active age, from lower social strata and with multibacillary forms of the disease. A total of 62% of the patients were born and raised in São Paulo at the time of diagnosis. The others were born in the northeastern states of Minas Gerais, Paraná, Mato Grosso do Sul, Goiás, and Rio de Janeiro; however, it was not possible to obtain information on how long they had lived in São Paulo. 

Thirteen of the 16-loci VNTR were polymorphic and included GTA9, TTC21, TA10, AT17, and TA18 with high polymorphism (HGDI > 0.64); Ac8b, Ac8a,18-8 were moderately polymorphic (HGDI between 0.35 and 0.53); and GGT5, 27-5, 6-7, 12-5, and AC9 presented low polymorphism (HGDI between 0.09 and 0.27). The repeats 6-3, 21-3, and 23-3 were invariable (HGDI = 0).

Some reports demonstrated that the inclusion of TA10 may be useful for a regional discriminatory HGDI lower than 0.8, which was recently observed in a study on molecular epidemiology in Rondonópolis, Mato Grosso, Midwest, Brazil (manuscript in preparation). This suggests that the HGDI of VNTRs is region-dependent and must be defined locally to determine the stringency of the cluster definition based on multiple-locus VNTR analysis (MLVA)^11^.

Of the 125 *M. leprae* isolates analyzed for cluster definition using 16-loci, 97 (77.6%) had unique profiles, whereas 28 (22.4%) belonged to 12 clusters, including 11 composed of two isolates each and one of six isolates. By using 13-loci to define genotypes (excluding TA18 [HGDI = 0.87], AT17 [HGDI = 0.81], and TA10 [HGDI = 0.74]), we observed the maintenance of the same isolates as of 16-loci; however, they were composed of more samples, with 73 (58.4%) isolates with a unique profile and 52 (41.6%) in cluster, composed of two, three, seven, 11, and 14 isolates, respectively. The clustering rates of the total isolates evaluated for the 13 and 16-loci were 32% and 13%, respectively.

Despite the genetic variability of *M. leprae*, 16 and 13 VNTR loci-based MLVA typing showed sufficient heterogeneity and stability to distinguish transmission clusters and link isolates belonging to recent transmission events, as reported earlier by Fontes *et al*. (2017)^11^.

SNP type 3 was observed in 121 isolates (97%) and SNP type 2 (Figure 2) in four (3%). Four samples of SNP type 2 were isolated from different regions of the state and all were diagnosed in 2012. 

**FIGURE 2:**
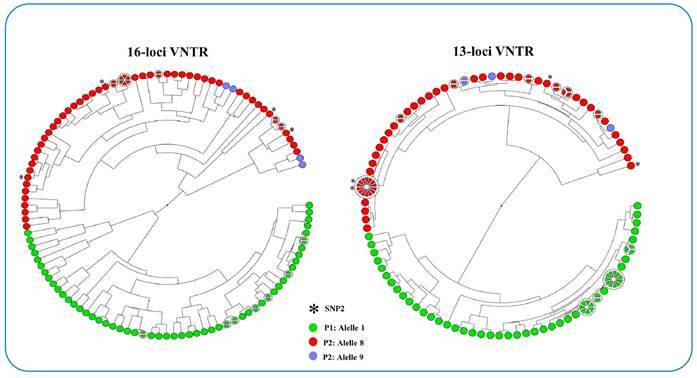
Population structure of *M. leprae* isolates based on 16 and 13-loci VNTR. Formation of two populations based on locus 18-8. (P1) red and lilac color represent 8 and 9 copies, respectively, and (P2) green 3 copies.

A study conducted by Monot *et al.* (2005)^5^ demonstrated that SNP analysis can be used to observe migratory flows that spread leprosy worldwide, and when combined with microbial phylogeography, can also help monitor the spread of microorganisms and the movement of their host. Regarding the analysis of the major SNP types in the region, in concordance with earlier studies in the southeast region of Brazil^12^, we observed the predominance of type 3 SNP, possible genotype 3I-2, and a few isolates with type 2. 

Since the original description of the four main SNP types, they have been further subdivided into 16 main SNP types. 3I-1, which would be relatively closer to the medieval European strains^5^; further investigation is warranted in the region of São Paulo to characterize the circulating SNP subtypes. Benjak *et. al.* (2018)^13^ reported that in the states of Rio de Janeiro and São Paulo, *M. leprae* especially harbored the genotype 3I-2, and a few strains from Sao Paulo also showed this genotype.

When analyzing the UPGMA-based tree considering 16 and 13-loci (excluding TA18, AT17, TA10), we observed the formation of two population-based on locus 18-8, where P1 was characterized by 8 and 9 copies and P2 by 3 copies (Figure 2). This pattern was disrupted only when 18-8 was excluded from the analysis.

When performing analysis of the genotypes, it was observed that they were distributed among both populations, despite the fact that isolates with SNP type 2 remained in P2. In Brazil, alleles with three copies are mostly present in isolates from the states of Rondônia, whereas alleles with eight and nine copies are present in isolates from Rio de Janeiro^12^. Alleles with eight copies have also been observed in countries such as Colombia^14^ and China^6^ ^,^ ^15^. The same pattern was observed in the population structure of Cebu (Philippines), which also had two groups separated by locus 18-8 (alleles with eight and four copies)^4^. 

Spatial analysis of clusters based on 13 and 16-loci VNTR loci was not performed because the clusters were formed by municipalities from different Departments of Health in São Paulo. However, two scenarios of intra and extrafamilial transmission, based on 16 loci and SNP, were detected (Figure 3). 

**FIGURE 3:**
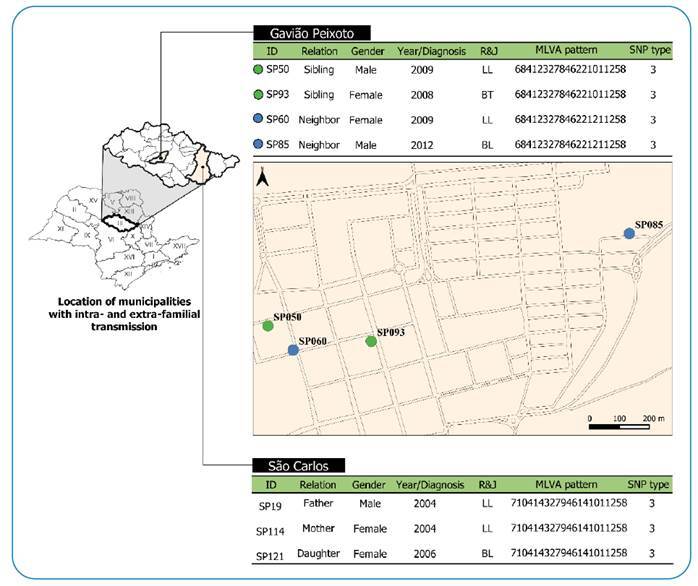
Georeferencing and clinical epidemiological data of two scenario intra- and extra-family transmission.

The first was formed by the father, mother, and daughter residing in the municipality of São Carlos. The second scenario comprised four patients residing in the city of Gavião Peixoto. Three patients lived in the same neighborhood, with an average distance of 245 m between them. Two of these individuals were siblings (SP 50 and SP 93). The SP 85 case lived in a neighborhood adjacent to the others, with an average distance of 1.141 m.

This study showed the important ongoing transmission of *M. leprae* isolates based on the distribution of genotypes with high similarity and the identification of family and social groups. Considering that São Paulo reached the goal of eliminating leprosy in 2006, these results indicate that there are a considerable number of cases circulating, and that combat and control actions must be reinforced and maintained in this Brazilian state, especially in a health department with low endemicity.

Despite including only 0.5% of the cases reported during the period in this study, we found members of the same family and neighbors in a cluster, showing the potential of this method to find links retrospectively.

We found high clustering levels considering the low representativeness of the sampling, with probably the largest timeframe study in leprosy typing studies. This proves that some genotypes are circulating in the population for considerable periods, that is, in the post-elimination period and as a result of the long incubation period of the disease.

Our findings strongly suggest that specific regions in São Paulo has had active and recent transmission of *M. leprae*; therefore, municipalities need to strengthen and expand strategic actions to interrupt the chain of transmission of the disease. 
